# Peroxisome Proliferator Activated Receptor Agonists Modulate Transposable Element Expression in Brain and Liver

**DOI:** 10.3389/fnmol.2018.00331

**Published:** 2018-09-19

**Authors:** Laura B. Ferguson, Lingling Zhang, Shi Wang, Courtney Bridges, R. Adron Harris, Igor Ponomarev

**Affiliations:** ^1^Waggoner Center for Alcohol & Addiction Research, The University of Texas at Austin, Austin, TX, United States; ^2^MOE Key Laboratory of Marine Genetics and Breeding, Ocean University of China, Qingdao, China; ^3^Laboratory for Marine Biology and Biotechnology, Qingdao National Laboratory for Marine Science and Technology, Qingdao, China

**Keywords:** transposable elements, retrotransposons, PPAR, psychiatric, gene expression

## Abstract

Peroxisome proliferator activated receptors (PPARs) are nuclear hormone receptors that act as transcription factors in response to endogenous lipid messengers. The fibrates and thiazolidinediones are synthetic PPAR agonists used clinically to treat dyslipidemia and Type 2 Diabetes Mellitus, respectively, but also improve symptoms of several other diseases. Transposable elements (TEs), repetitive sequences in mammalian genomes, are implicated in many of the same conditions for which PPAR agonists are therapeutic, including neurodegeneration, schizophrenia, and drug addiction. We tested the hypothesis that there is a link between actions of PPAR agonists and TE expression. We developed an innovative application of microarray data by mapping Illumina mouse WG-6 microarray probes to areas of the mouse genome that contain TEs. Using this information, we assessed the effects of systemic administration of three PPAR agonists with different PPAR subtype selectivity: fenofibrate, tesaglitazar, and bezafibrate, on TE probe expression in mouse brain [prefrontal cortex (PFC) and amygdala] and liver. We found that fenofibrate, and bezafibrate to a lesser extent, up-regulated probes mapped to retrotransposons: Short-Interspersed Elements (SINEs) and Long-Interspersed Elements (LINEs), in the PFC. Conversely, all PPAR agonists down-regulated LINEs and tesaglitazar and bezafibrate also down-regulated SINEs in liver. We built gene coexpression networks that partitioned the diverse transcriptional response to PPAR agonists into groups of probes with highly correlated expression patterns (modules). Most of the differentially expressed retrotransposons were within the same module, suggesting coordinated regulation of their expression, possibly by PPAR signaling. One TE module was conserved across tissues and was enriched with genes whose products participate in epigenetic regulation, suggesting that PPAR agonists affect TE expression via epigenetic mechanisms. Other enriched functional categories included phenotypes related to embryonic development and learning and memory, suggesting functional links between these biological processes and TE expression. In summary, these findings suggest mechanistic relationships between retrotransposons and PPAR agonists and provide a basis for future exploration of their functional roles in brain and liver.

## Introduction

Peroxisome proliferator activated receptors (PPARs), members of the nuclear hormone receptor family, are ligand-activated transcription factors. There are three PPAR isotypes: PPARα, PPARγ and PPARβ (sometimes called PPARδ) with distinct expression patterns, tissue distribution, and ligand specificity (Grygiel-Gorniak, 2014). PPARα is most highly abundant in metabolically active tissues including liver, heart, intestine, and kidney, PPARγ is most abundant in adipose tissue, and PPARβ/δ is ubiquitously expressed, often at relatively higher levels than PPARα or PPARγ (Kliewer et al., 1994; Braissant et al., 1996). All PPAR isotypes are expressed in brain, including the prefrontal cortex (PFC) and amygdala, and appear to be most highly expressed in neurons (Moreno et al., 2004; Warden et al., 2016). Their main function is to regulate fatty acid metabolism but also have roles in inflammation, glucose metabolism, adipogenesis and myelination [for review, see (Tyagi et al., 2011)]. Fibrates are PPARα agonists used to treat dyslipidemia, thiazolidinediones (TZDs) are PPARγ agonists used to treat type 2 diabetes, and the glitazars are dual PPARα and PPARγ agonists used to treat dyslipidemia and type 2 diabetes comorbidities. Although PPARβ /PPARδ agonists are not used clinically, they have been used as performance enhancers by athletes and have been prohibited by the World Anti-Doping Agency.

Transposable elements (TEs) are repeating genomic elements that have the ability to translocate across the genome. First discovered by Barbara McClintock in maize in the 1940s, TEs have been found across different taxonomic groups. They can have both beneficial and deleterious effects on the host. They can drive diversity and evolution through their role as controlling elements that can lead to novel regulation of genes, but they can also drive disease through their role as insertional mutagens [for review, see (Reilly et al., 2013; Chuong et al., 2017)]. Many TEs are conserved across species including fruit flies, rodents and humans, where they are estimated to make up almost half of the genome (Adams et al., 2000; Nellaker et al., 2012). TEs can be divided into two major classes: DNA transposons that translocate via a cut-and-paste method (but are not mobile in mammals), and retrotransposons that translocate via a copy-and-paste method using an RNA intermediate. Retrotransposons can be further subdivided into categories based on whether or not they contain Long Terminal Repeats (LTRs, e.g., endogenous retroviruses - ERVs). Retrotransposons lacking LTRs include Long-Interspersed Elements (LINEs), Short-Interspersed Elements (SINEs), Alu and SVA elements. For more information on different classes of TEs the interested reader is referred to reviews (Muotri et al., 2007; Reilly et al., 2013; Chuong et al., 2017).

Although representing a large portion of the genome in different species, most TE sequences are non-functional and cannot move in the genome. The host uses sophisticated epigenetic mechanisms to create highly condensed heterochromatin to regulate TE expression (Slotkin and Martienssen, 2007; Maze et al., 2011; Ponomarev et al., 2012; Xie et al., 2013; Du et al., 2016). Inhibition of retrotransposon expression occurs through extensive DNA methylation at retrotransposon promoter sequences [reviewed in (Yoder et al., 1997)]. However, retrotransposons can be expressed when the epigenetic silencing is released (Slotkin and Martienssen, 2007). For example, one study linked hypomethylated genomic areas with increased expression of LTRs in human alcoholic frontal cortex compared to controls (Ponomarev et al., 2012), another study linked hypomethylation with increased activation of ERVs in mouse tumors (Howard et al., 2008), and another found that cocaine-mediated reductions of a heterochromatic marker (H3K9me3) were associated with increased LINE-1 expression in mouse nucleus accumbens (Maze et al., 2011).

Transposable elements have been implicated in many pathological conditions, including approximately 100 single-gene diseases (Hancks and Kazazian, 2012), such as hemophilia A (Sukarova et al., 2001; Ganguly et al., 2003; Green et al., 2008), and cystic fibrosis (Chen et al., 2008). Retrotransposons (ERVs and LINE1 elements) have also been linked to more complex diseases including diabetes (Pascual et al., 2001; Dickerson et al., 2008), cancers [for review, see (Burns, 2017)], and several psychiatric disorders (Guffanti et al., 2014), such as alcohol and cocaine addiction (Maze et al., 2011; Ponomarev et al., 2012), autism (Balestrieri et al., 2012), Attention Deficit Hyperactivity Disorder (Balestrieri et al., 2014), depression (Weis et al., 2007), schizophrenia (Yolken et al., 2000; Karlsson et al., 2001, 2004; Frank et al., 2005; Weis et al., 2007; Dickerson et al., 2008; Perron et al., 2008, 2012a,b; Yao et al., 2008; Huang et al., 2011; Lin et al., 2011; Bundo et al., 2014; Slokar and Hasler, 2015), bipolar disorder (Yolken et al., 2000; Weis et al., 2007; Frank et al., 2005; Perron et al., 2012b), post-traumatic stress disorder (Ponomarev et al., 2010; Rusiecki et al., 2012), Rett syndrome (Muotri et al., 2010), and neurodegeneration (Cartault et al., 2012; Li et al., 2012). Besides their clinical uses for dyslipidemia and diabetes, PPAR agonists have shown therapeutic potential (mostly preclinical evidence) for several brain diseases including neurodegeneration (Bordet et al., 2006; Barbiero et al., 2014; Fidaleo et al., 2014; Avagliano et al., 2016; Dickey et al., 2016; Makela et al., 2016; Zhou et al., 2016; Wang Y. et al., 2017), mood disorders (Kemp et al., 2014; Ji et al., 2015; Scheggi et al., 2016; Colle et al., 2017; Jiang et al., 2017; Li et al., 2018; Ni et al., 2018), schizophrenia (Rolland et al., 2012), and several types of substance use disorders (Melis et al., 2010; Stopponi et al., 2011; Panlilio et al., 2012, 2013; Le Foll et al., 2013; Stopponi et al., 2013; Ferguson et al., 2014; Karahanian et al., 2014; Blednov et al., 2015; de Guglielmo et al., 2015; Haile and Kosten, 2017; Rivera-Meza et al., 2017). Therefore, PPAR agonists and TEs are clinically related, i.e., are associated with similar diseases. The rationale for this study is based on this clinical relatedness; we reasoned that because PPAR agonists and TEs are associated with many of the same diseases that their mechanisms of action could be interacting. Here, we tested the hypothesis that activation of PPARs regulates transcriptional activity of TEs. We mapped Illumina microarray probes to genomic locations that correspond to different classes of TEs and used this information to study the effects of PPAR agonists on expression of TEs in the amygdala, PFC, and liver of male C57Bl/6J mice.

## Materials and Methods

### Animals and Drug Administration

We previously measured the effects of peripherally administered PPAR agonists on brain and liver gene expression (Ferguson et al., 2014). Briefly, we administered (p.o.) saline or one of three PPAR agonists: 150 mg/kg of the PPARα agonist fenofibrate (feno), 75 mg/kg of the pan-PPAR agonist bezafibrate (beza) or 1.5 mg/kg of the dual PPARα-PPARγ agonist tesaglitazar (tesa) to male C57Bl/6J mice (8–12 weeks old, *n* = 10 per group) once per day for 8 days. See (Ferguson et al., 2014) for details. All experiments were approved by The University of Texas at Austin Institute for Animal Care and Use Committee and were conducted in accordance with NIH guidelines with regard to the use of animals in research.

### Gene Expression

Twenty-four hours after the last administration of PPAR agonist, amygdala, PFC, and liver were harvested. We extracted, amplified, and biotin-labeled total RNA and sent aliquots of labeled cRNA to the Yale Center for Genome Analysis, where they were hybridized to Illumina^®^ MouseWG-6 v2 Expression BeadChips. See (Ferguson et al., 2014) for details. Microarray data were deposited in the NCBI Gene Expression Omnibus (GEO) database under accession number GSE67796.

### Transposable Element (TE) Annotation

To identify microarray probes targeting potential TE regions, we first obtained probe sequences of all Illumina probes from the probe annotation file MouseWG-6_V2_0_R3_11278593_A^1^ and mapped them to mouse genome (NCBI37/mm9) downloaded from the University of California, Santa Cruz (UCSC) Genome Bioinformatics website^2^ using Short Oligonucleotide Analysis Package (Li et al., 2009). Then we compared the probe genomic coordinates with the coordinates of TEs reported in the Repeat Masker table downloaded from UCSC Genome Bioinformatics website. A probe was considered to be targeting a TE region only if it falls completely within an annotated TE region. We constructed four TE data sets covering the major TE classes: DNA transposons, long-term repeat (LTR), long interspersed nuclear element (LINE), and short interspersed nuclear elements (SINE). **Supplementary Table S1** contains the list of Illumina probes mapped to each TE class.

### Statistical and Gene Network Analyses

Data from each tissue were analyzed separately. Variance stabilization transformation and quantile normalization were used to pre-process the data using the Bioconductor Lumi package (Du et al., 2008). Groups being compared were normalized together, i.e., saline and bezafibrate, saline and tesaglitazar, and saline and fenofibrate. Outlier values for each gene within a group were removed using Grubbs’ test (*p* < 0.05). To detect genes differentially expressed between treated and control mice, we used the Bioconductor Limma package to fit a linear model for each gene (Ritchie et al., 2015). This analysis generated a *t*-value for each probe, with a positive *t*-value indicating up- and a negative *t*-value indicating a down-regulation after drug treatment.

We conducted weighted gene co-expression network analysis (WGCNA) as previously described (Ferguson et al., 2014). Briefly, all reliably detected genes were included in the network construction for the gene network analysis. Data from all treatments (fenofibrate, tesaglitazar, bezafibrate and saline) were used to detect co-expression patterns. Signed networks were constructed using R and custom functions available at https://horvath.genetics.ucla.edu/html/CoexpressionNetwork/Rpackages/WGCNA/. The power was set to β = 11, β = 14 and β = 7 for the amygdala, PFC and liver network, respectively, and minimum module size of 100, 150, and 100 genes for amygdala, PFC and liver, respectively. A dendrogram cut height of 0.99 was used for amygdala and PFC, and 0.995 was used for liver. Similar modules were merged to reduce the number of modules used for analyses. Gene modules corresponding to branches of the dendrogram were labeled in unique colors. Genes whose profile failed to cluster in the network were labeled in gray.

### Bioinformatics Analysis

Hypergeometric test was performed to assess TE enrichment for each set of differentially expressed genes or gene network module and Benjamini-Hochberg’s FDR was calculated to correct for multiple tests (except where noted otherwise). We plotted the *t*-value distribution of each TE class and used a one-sample, two-tailed t-test to determine if the average *t*-values for those TE classes were different from 0 (Bonferroni-corrected *p*-value < 0.05 was the threshold we used for statistical significance).

For functional annotation of the TE-enriched modules, we used the hypergeometric test to assess enrichment of cell types [neurons, oligodendrocytes, microglia, astrocytes, hepatocytes, Kupffer cells, and hepatic stellate cells (HSCs) (Sugino et al., 2006; Takahara et al., 2006; Cahoy et al., 2008; Oldham et al., 2008)] and the PPAR agonist-regulated genesets (i.e., genes differentially expressed at a nominal *p*-value < 0.05 after PPAR agonist treatment) as previously described (Ferguson et al., 2014). We curated the list of ethanol consumption-related genes from the literature on mutant mouse data (gene knockdown or overexpression). More information can be found in these reviews (Crabbe et al., 2006; Mayfield et al., 2016). Because cells use epigenetic mechanisms to control TEs (See Introduction), we looked for enrichment of 171 genes whose encoded proteins are involved in epigenetic chromatin remodeling and modification in the TE-enriched modules (attained from the SABiosciences website; **Supplementary Table S2**).

### Comparison of Tissue Networks

We built and analyzed co-expression networks for each tissue individually and compared the co-expression patterns of each tissue’s network. The hypergeometric distribution was used to assess the significance of internetwork module overlap. We used Cytoscape 3.2.1 (Shannon et al., 2003) to visualize the network comparisons (Killcoyne et al., 2009). Only conservative modules with overlap *p* < 10^-3^ are presented. The 13,623 probes that were common between amygdala, PFC, and liver networks were used in the network comparison. To determine the enriched pathways, phenotypes and ontologies of the genes in the conserved TE module we used WebGestalt^3^ (Wang J. et al., 2017). We used the criteria that the enriched category must include ≥ 3 genes and pass significance threshold of FDR-adjusted hypergeometric *p* < 0.05. Phenotype data are derived from literature, knockout data, and other sources curated by Mouse Genome Informatics.

### qRT-PCR Validation

We validated Illumina probe ILMN_1244099 (which corresponds to ORF1 of a LINE1 element). We designed the probe in such a way as to detect the LINE1 elements, specifically. Tissue and total RNA isolation were described previously (Ferguson et al., 2014). PFC cDNA was synthesized with the iScript Select cDNA Synthesis Kit (BIORAD, priming strategy: oligo(dT) primers) for 37 samples (due to limited RNA quantity, 3 PFC samples could not be included). Sample information can be found on GEO accession GSE67796. Primer Express was used to design a Taqman assay to detect the LINE1 TE that contained the ILMN_1244099 probe sequence. Gapdh was used as an endogenous control. qBase Plus software (Biogazelle, Zwijnaarde, Belgium) was used for data analysis. A two-sided Mann Whitney test was used to assess the significance of the between group differences.

Taqman Assay [45–108] amp. length of 64

Forward Primer: CCAGAAGAGCCTGGACAGATG

Reverse primer: AGTAGCCTGGGCTGGCATT

Probe: TATACAGACACTAAGAGAACAC

## Results

### TE Mapping

We previously studied the organization of brain transcriptome in human alcoholics and discovered that TEs, such as LTRs and SINEs, are co-expressed and regulated in alcoholics (Ponomarev et al., 2012). Here, we performed TE enrichment analysis to determine if a TE regulation also exists in mouse, and if PPAR agonist treatment has any effect on TE regulation. Out of 45,281 probes on the microarray, a total of 2,071 probes were found to target TEs, including 104 DNA transposons, 504 LINEs, 576 LTRs and 887 SINEs (**Supplementary Tables S1, S4**). Overrepresentation analysis indicated that the probes mapped to TEs contained a disproportionate number of unannotated genes, supporting our mapping method (**Supplementary Figure S1**).

### PPAR Agonists Increase TE Expression in the PFC and Decrease TE Expression in the Liver

We previously characterized brain and liver gene expression profiles following an 8-day, treatment with PPAR agonists (Ferguson et al., 2014). Here, we used the aforementioned mapping information to study TE expression in the PPAR agonist-regulated genesets (we define PPAR agonist-regulated genes as those that were differentially expressed between PPAR agonist and saline treatment at *p* < 0.05). We used the hypergeometric test to determine if more TEs were in the PPAR agonist-regulated genesets than chance level and used Bonferroni corrected *p* < 0.05 as a statistical threshold. We found that SINEs and LINEs were enriched in the genesets that are up-regulated by fenofibrate in the PFC and SINEs are enriched in the genesets up-regulated by bezafibrate in the PFC (**Table 1**). Conversely to the overall upregulation of TEs observed in PFC, LINEs were enriched in the genesets down-regulated by bezafibrate and tesaglitazar in liver (**Table 1**). TEs were not enriched in the genesets regulated by PPAR agonists in amygdala (**Supplementary Table S3**).

**Table 1:** Transposable elements (TEs) in PPAR regulated genesets.

	PFC			Liver		
	Beza	Feno	Tesa	Beza	Feno	Tesa
SINE (all DEGs)	n.s.	**3.92E-09**	n.s.	n.s.	n.s.	n.s.
SINE (up-regulated genes)	**2.68E-05**	**2.22E-22**	n.s.	n.s.	n.s.	n.s.
SINE (down-regulated genes)	n.s.	n.s.	n.s.	n.s.	n.s.	n.s.
LINE (all DEGs)	n.s.	**2.70E-05**	n.s.	n.s.	n.s.	n.s.
LINE (up-regulated genes)	n.s.	**1.10E-15**	n.s.	n.s.	n.s.	n.s.
LINE (down-regulated genes)	n.s.	n.s.	n.s.	**4.02E-06**	6.80E-03	**3.81E-04**
LTR (all DEGs)	n.s.	n.s.	n.s.	n.s.	n.s.	n.s.
LTR (up-regulated genes)	n.s.	2.17E-02	n.s.	n.s.	n.s.	n.s.
LTR (down-regulated genes)	n.s.	n.s.	n.s.	n.s.	n.s.	n.s.
DNA (all DEGs)	n.s.	n.s.	n.s.	n.s.	n.s.	n.s.
DNA (up-regulated genes)	n.s.	n.s.	n.s.	n.s.	n.s.	n.s.
DNA (down-regulated genes)	n.s.	n.s.	n.s.	n.s.	n.s.	n.s.


*We previously characterized brain and liver gene expression profiles following an 8-day, systemic treatment with PPAR agonists (Ferguson et al., 2014). Here, we characterized TE expression in the PPAR-regulated genesets (PPAR-regulated genes are those that were differentially expressed between PPAR agonist and saline treatment at *p* < 0.05). We used the hypergeometric test to determine if PPAR agonists regulated TEs more than chance level and used Bonferroni corrected *p* < 0.05 as a statistical threshold. In the table, bold *p*-values are those that were significant at this threshold and non-bold *p*-values are those just below this threshold. We looked at enrichment in all differentially expressed genes (DEGs), up-regulated genes, and down-regulated genes as denoted by row name.*

**FIGURE 1:**
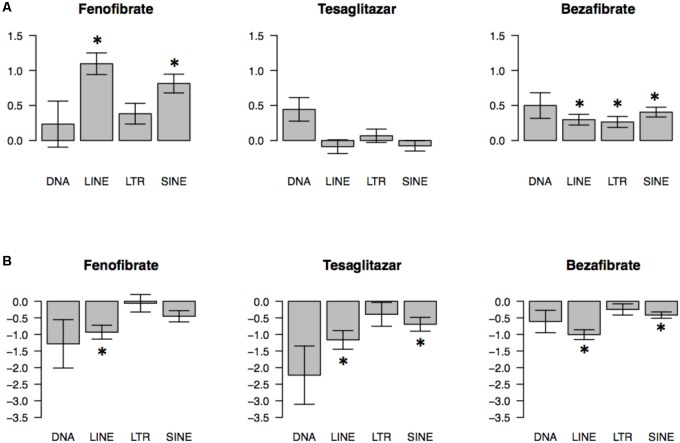
Global effects of PPAR agonists on transcriptomes of 4 classes of transposable elements (TEs) in mouse prefrontal cortex (PFC) **(A)** and liver **(B)**. Direction and magnitude of PPAR agonist-induced changes were estimated by plotting *t* distributions for probes that map to a specific TE type. A *t*-value represents the magnitude and direction of PPAR agonist effects (*t* < 0 indicates downregulation; *t* > 0 indicates upregulation by PPAR agonist). The barplots show the average ± SEM *t* values (^∗^*p* < 0.05; based on one-sample *t*-test comparing average *t*-values to zero chance with Bonferroni’s correction). *N* = 7–10 per group.

To examine general tendencies of each TE class to be regulated by PPAR agonists, we used an effect size approach and estimated shifts of the *t*-value distribution of each TE class in PFC and liver. This analysis confirmed that SINEs and LINEs were up-regulated by fenofibrate and LINEs were up-regulated by bezafibrate in PFC (**Figure 1**). It also suggested that SINEs and LTRs were generally up-regulated by bezafibrate because the average *t*-values for those TE classes were statistically greater than 0 (*p*-value < 0.05), though this was a relatively weak effect. For liver, this method revealed that LINEs were down-regulated by fenofibrate, tesaglitazar, and bezafibrate. Also, SINEs were down-regulated by tesaglitazar and bezafibrate.

**FIGURE 2:**
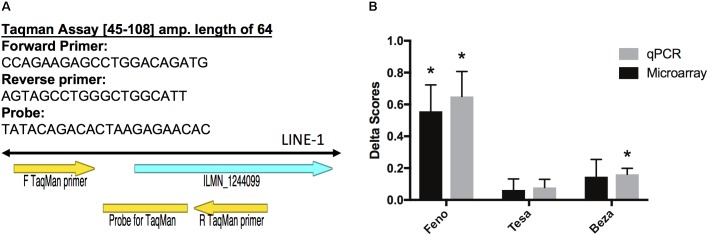
Validation of microarray results. **(A)** Primer Express software was used to design Taqman primers and probe sequences to detect the LINE-1 element that corresponds to Illumina probe ILMN_1244099 (left). Delta scores were calculated by subtracting the normalized saline group average from each of the normalized gene expression values for each treatment group. **(B)** Error bars represent the standard error of the mean. The asterisks represent significant differences at *p* < 0.05 between treatment and saline groups as assessed by the limma moderated *t* statistic for the microarray group comparisons (Ritchie et al., 2015), and a two-sided Mann Whitney test for the qPCR group comparisons. *N* = 7–10 per group.

We used qPCR to validate some of our microarray findings and specifically measure expression of the LINE1 TE that was originally detected by the ILMN_1244099 probe (**Figure 2**). This probe was up-regulated in PFC by fenofibrate and we confirmed this up-regulation with the qPCR assay (group difference *p* = 8.23E-05) (**Figure 2**). Moreover, qPCR detected a significant difference in the levels of the LINE1 element between the bezafibrate and saline treatment groups that the microarray was not sensitive enough to detect (*p* = 0.01) (**Figure 2**). The correlation between the microarray and qPCR data was 0.67 (*p* < 0.0001).

### Effects of PPAR Agonists on TE-Enriched Gene Co-expression Networks

**FIGURE 3:**
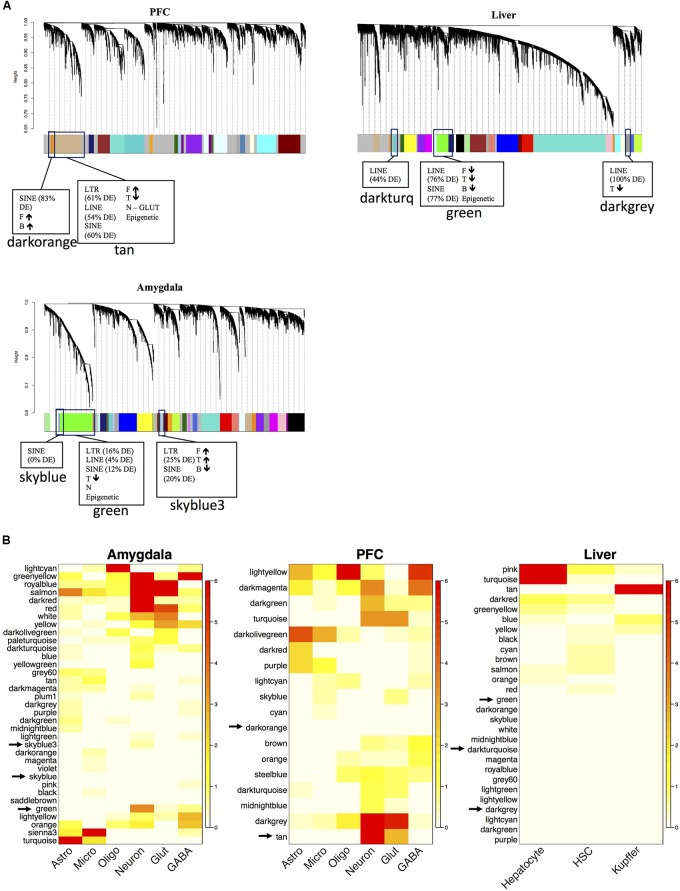
Network analysis of the mouse transcriptome and functional annotation of gene modules after PPAR agonist administration. The dendrogram of the gene network that was constructed for each tissue separately (*N* = 7–10 per group) **(A)** The x-axis corresponds to genes detected on the microarray and the y-axis to the co-expression distance between genes determined by the extent of topological overlap. Dynamic tree cutting identified modules, generally dividing them at significant branch points in the dendrogram. Genes in the modules are color-coded. Genes not assigned to a module are labeled gray. The TE-enriched modules are designated by boxes. The adjacent parentheses indicate the percentage of the respective TE class in the module that is differentially expressed (DE). If more PPAR-agonist regulated genes are in the module than expected by chance, it is indicated in the box. The arrows indicate the direction of fold-change induced by the PPAR agonist for the PPAR agonist-regulated genes in that module. F, fenofibrate, T, tesaglitazar, and B, bezafibrate. Heatmap plots of the hypergeometric *p*-values from the over-representation (enrichment) analysis for the differentially expressed genes (DEGs) and cell type-specific genes **(B)**. Each row in the heatmap corresponds to one module (labeled by color on the left) and each column in the heatmap corresponds to the category being tested for over-representation. Scale bar on the right represents –log(hypergeometric *p*-values) used to assess statistical significance of over-representation (red, high statistical significance). Rows were arranged by hierarchical clustering. Analysis conducted and graphs made in R.

We built gene-gene co-expression networks to parse the diverse transcriptional response to PPAR agonist administration in brain and liver. Network construction revealed several groups of highly correlated genes (i.e., modules), and we determined that microarray probes corresponding to TEs were enriched in several of these modules (**Figure 3**). Interestingly, most of the TE-enriched modules were also enriched with PPAR agonist-regulated genes (**Figure 3** and **Supplementary Table S5**). These findings suggest that PPAR agonists regulate expression of TEs in a coordinated manner. The percentage of PPAR agonist-regulated TEs within TE-enriched modules was higher for PFC and liver than for amygdala (**Figure 3**). Confirming and extending possible PPAR agonist-specific and tissue-specific effects observed in the differential expression results; fenofibrate and tesaglitazar differentially regulated TEs in a TE-enriched module (tan) in PFC (fenofibrate increased while tesaglitazar decreased TEs) and fenofibrate differentially regulated TEs in the TE-enriched modules in PFC (tan and darkorange) and liver (green) (fenofibrate increased TEs in PFC and decreased in liver).

Because epigenetic modifications are an important mechanism the host uses to control and suppress transposition events, we looked for enrichment of genes involved in epigenetic functions within the modules. One TE-enriched module in each tissue was also enriched with epigenetic genes. In PFC, the tan module was enriched with epigenetic genes (**Table 2**) and also SINEs, LINEs, LTRs, glutamatergic neuronal genes, fenofibrate up-regulated genes, and tesaglitazar down-regulated genes (**Figure 3A**). In liver and amygdala, the green modules were enriched with epigenetic genes (**Table 2**). The green module in the amygdala was also enriched with SINEs, LINEs, LTRs, neuronal genes, and tesaglitazar down-regulated genes (**Figure 3A**). The green module in the liver was also enriched with SINEs, LINEs, LTRs, and the down-regulated genes of each PPAR agonist (fenofibrate, tesaglitazar, and bezafibrate) (**Figure 3A**). PPAR-mediated regulation of epigenetic genes in TE-enriched modules points to potential mechanisms underlying the effects of PPAR agonists on TE expression.

**Table 2:** Enrichment of genes whose products are involved in epigenetic functions within network modules.

Tissue	Module	*P*-Value	# Epigenetic Genes in Module	Epigenetic genes in module
PFC	Tan	1.12E-04	32	**Actb**, **Ash1l**, Atf2, Baz2a, **Brd1**, **Brd2**, Brpf1, Brwd3, **Cbx5**, **Chd1**, **Chd2**, **Chd4**, Chd5, **Chd7**, Dot1l, Ehmt1, Ezh2, **Hdac3**, Ing3, Mbd4, **Mysm1**, **Nsd1**, **Pcgf5**, Prmt5, Rps6ka5, Setd2, Setd8, **Smarca2**, **Suz12**, Ube2b, Usp22, **Whsc1**
Liver	Green	2.64E-03	14	**Actb**, **Ash1l**, Baz2a, **Brd2**, **Brd3**, **Brd4**, **Chd1**, **Chd2**, **Chd4**, **Ehmt1**, Kat2b, **Mysm1**, **Nsd1**, Suz12
Amygdala	Green	1.73E-04	36	Actb, Ash1l, Baz2a, **Brd1**, Brd2, Brd4, Brd8, Brpf1, Cbx3, Cbx5, Chd1, Chd2, **Chd4**, Chd5, Chd7, Chd9, Csrp2bp, Dot1l, **Ehmt1**, Esco1, Ezh2, Hdac2, **Hdac3**, Ing3, Mbd4, Mysm1, Nsd1, Pcgf5, **Prmt5**, Setd2, Setd8, **Smarca2**, Spen, Suz12, Usp22, Whsc1


*One of the TE-enriched modules in each tissue was also enriched with epigenetic genes. These modules were conserved across tissues, meaning that these 3 modules were comprised of similar genes in amygdala, PFC, and liver (see text for details). In PFC, the tan module was enriched with epigenetic genes and also SINES, LINES, and LTRs, glutamatergic neuronal genes, fenofibrate up-regulated genes, and tesaglitazar down-regulated genes. In liver and amygdala, the green modules were also enriched with epigenetic genes. Amygdala green was enriched with SINES, LINES, LTRs, neuronal genes, and tesaglitazar down-regulated genes. Liver green was enriched with SINES, LINES, LTRs, and the down-regulated genes of each PPAR agonist (fenofibrate, tesaglitazar, and bezafibrate). Bolded genes are those in the module that are differentially expressed after PPAR agonist treatment.*

### Preservation of TE-Enriched Modules Across Tissues

**FIGURE 4:**
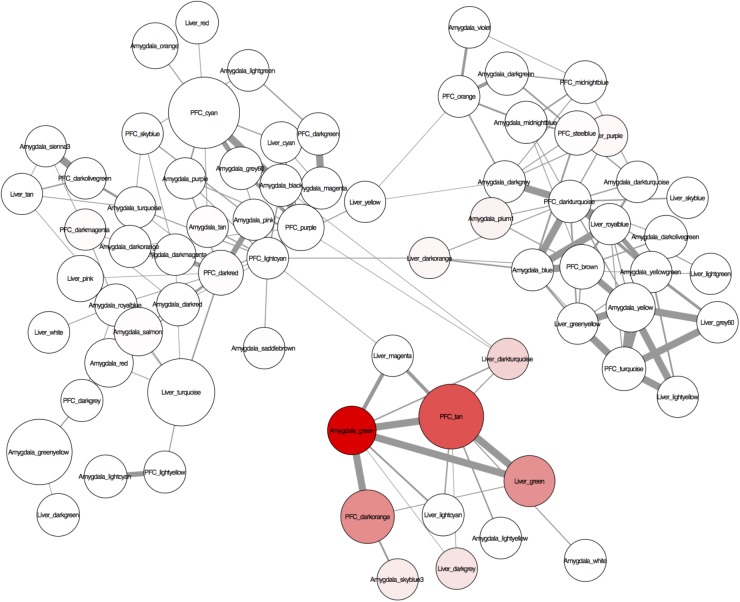
A visualization of the conserved regulation of gene expression networks across the tissues analyzed (liver, amygdala, and PFC) created with Cytoscape 3.2.1. The nodes are modules of the individually constructed networks for each tissue (see **Figure 3** for details). Only probes common to all tissues were used to assess module overlap. The edges are the –log(hypergeometric *p*-values) of the number of overlapping probes between 2 nodes (modules). Edge thickness represents the significance of the overlap between the nodes (modules) – the thicker the line, the more significant the overlap. The nodes (modules) were assessed for enrichment of transposable elements (TEs) and genes differentially expressed at *p* < 0.05 after systemic treatment with one of 3 PPAR agonists (fenofibrate, tesaglitazar, or bezafibrate) (see methods). Node color represents enrichment with TEs (higher intensity = more enrichment). Node size represents enrichment with PPAR agonist-regulated genes (the larger the node, the more enriched with PPAR agonist-regulated genes).

To identify conserved patterns of TE expression, we assessed the overlap of gene co-expression modules between the three separate tissue-specific gene networks. As expected, the two brain networks had more conserved modules between them than between the brain and liver networks (**Supplementary Figure S2**). The TE modules from the various tissues form a distinct cluster within the network and are highly overlapping across tissues (red nodes in **Figure 4**). Interestingly, several of the TE modules were responsive to PPAR agonists, i.e., enriched with DEGs (node size in **Figure 4**), suggesting a specific role for PPAR agonists in regulation of TEs. The tan TE module in PFC was more regulated by PPAR agonists (i.e., larger node size) than the green TE modules in liver and amygdala, supporting our finding that TEs are highly enriched in the PPAR-regulated genesets in PFC.

There was a highly significant overlap between the tan module in PFC, green module in liver and green module in amygdala (**Figure 4** and **Supplementary Figures S2, S3**). These modules share an enrichment of LINEs, SINEs, tesaglitazar down-regulated genes, neuronal genes (amygdala and PFC only) and genes known to be involved in epigenetic mechanisms (**Figure 3** and **Table 2**). There were 348 Illumina probes that were common between these 3 modules. We entered these into WebGestalt (Wang J. et al., 2017) to determine the enriched pathways, phenotypes, and ontologies. Of the 348 Illumina probes, 149 were used for analysis, as most probes mapped to TEs and therefore are unannotated and unable to be used for analysis. Two biological process ontologies (histone binding and protein complex scaffold), 2 KEGG pathways (Wnt signaling and adherens junction), and 1 Panther pathway (Cadherin signaling) emerged from this analysis (**Table 3** and **Supplementary Table S6**). Thirteen phenotypes were enriched in the conserved TE module that fell into 3 main categories: learning and memory, craniofacial morphology, and lethality (**Table 3** and **Supplementary Table S6**).

**Table 3:** Enriched functional categories in the conserved TE module.

Name	Number Genes	*P*-Value	FDR
Histone binding	11	4.74E-05	5.54E-03
Protein complex scaffold	7	4.63E-05	5.54E-03
Cadherin signaling pathway	5	1.74E-04	1.97E-02
Wnt signaling pathway - Mus musculus (mouse)	9	4.76E-05	1.41E-02
Adherens junction - Mus musculus (mouse)	7	1.36E-04	2.01E-02
Abnormal temporal memory	10	1.26E-05	4.48E-02
Abnormal associative learning	12	3.41E-05	4.48E-02
Abnormal contextual conditioning behavior	8	1.11E-04	4.48E-02
Abnormal nasal bone morphology	7	2.69E-05	4.48E-02
Abnormal craniofacial morphology	30	1.07E-04	4.48E-02
Craniofacial phenotype	30	1.07E-04	4.48E-02
Short nasal bone	5	1.09E-04	4.48E-02
Preweaning lethality	93	4.98E-05	4.48E-02
Embryonic lethality during organogenesis, complete penetrance	28	5.91E-05	4.48E-02
Pale yolk sac	93	6.11E-05	4.48E-02
Embryonic lethality during organogenesis	32	8.64E-05	4.48E-02
Abnormal neural tube closure	16	9.77E-05	4.48E-02
Lethality during fetal growth through weaning	48	1.00E-04	4.48E-02


*Gene-gene coexpression network analysis revealed a group of genes with tightly correlated expression levels which contained a high number of TEs (see text for details on network construction). Roughly this same group of correlated genes and TEs was identified in prefrontal cortex, amygdala, and liver of male C57Bl/6J mice. We submitted the annotated genes within this conserved TE module to WebGestalt (http://www.webgestalt.org) (Wang J. et al., 2017) to determine the enriched pathways, phenotypes and ontologies. Of the 348, 149 Illumina probes were used for analysis, as most probes were mapped to TEs and therefore unannotated and unable to be used for analysis. We used the criteria that the enriched category must include ≥ 3 genes and pass significance threshold of FDR-adjusted hypergeometric *p* < 0.05. The enriched category is given under the Name column. The number of unique genes contributing to the categorical enrichment and the *p*-value and FDR value are also provided in the table.*

Cell type is a major contributor to variability in gene expression datasets when whole tissue is analyzed (e.g., Oldham et al., 2006, 2008; Winden et al., 2009; Kang et al., 2011; Grange et al., 2014; Hawrylycz et al., 2015), which is the case here as the samples were from total homogenate. Gene coexpression network analysis, in combination with cell type specific datasets, reliably identifies gene networks specific for individual cell types (e.g., Winden et al., 2009; Grange et al., 2014). We identified that the TE-enriched conserved modules were associated with neurons in PFC and amygdala, and the liver module was not associated with any cell type (**Figure 3B**). This suggests that the effects of PPAR drugs on TE expression in brain are limited to neurons.

## Discussion

Peroxisome proliferator activated receptor agonists have demonstrated therapeutic potential for a range of diseases that include neuropsychiatric states. Many brain disorders are associated with up-regulation of TEs and little is known about TE function in brain, let alone the pharmacological regulation of TE expression patterns. We developed an innovative application of microarray data by mapping array probes to areas of the mouse genome that contain TEs. This enabled us to observe the effects of PPAR agonist administration on the expression of 4 TE classes in mouse brain and liver and make novel hypotheses regarding TE behavior in these tissues. Given the widespread clinical use of PPAR agonists, these findings have potential implications for a significant proportion of the population.

There are a number of ways that TEs can affect biological functions. For example, TEs can modify genomic architecture via insertional mutagenesis or post-insertional rearrangements [reviewed in (Goodier and Kazazian, 2008; Guffanti et al., 2014)]. TEs can also regulate the expression of other genes by introducing transcription start sites, inducing heterochromatization, or interfering with transcription via intron retention, exonization, or polyadenylation [reviewed in (Cowley and Oakey, 2013; Guffanti et al., 2014)]. Although LINEs are more mobile in brain versus other somatic tissue (e.g., skin) (Garcia-Perez et al., 2016), retrotransposition events are relatively rare, estimated to occur in caudate and cortical human neurons (postmortem) or neural progenitor cells (*in vitro*) at a rate of 1 in 100 to 1 in 10,000 insertion events per neuron or neural progenitor (Coufal et al., 2009; Evrony et al., 2012). Therefore, it is unlikely that the differentially expressed TEs we observed are retrotransposed and inserted into the genome, and it is still unclear if new insertions can occur in postmitotic neurons. Although our findings are observational, and the downstream effects of the changes in TE expression after PPAR agonist treatment and the exact molecular mechanisms by which PPAR agonists modulate TE expression remain unknown, these findings suggest a novel relationship between TEs and PPAR agonists.

Gene-gene coexpression network analysis clusters genes that perform similar biological tasks or act in similar pathways, which allowed us to propose “guilt-by-association” relationships and to hypothesize either regulation or/and functional roles of TEs based on the known functions of other genes within the same module. It is important to note that enrichment analyses are used to generate hypotheses, and future experiments are required to determine how PPAR agonists regulate TE expression and any potential downstream consequences of the observed TE expression changes. In addition to being enriched with probes that are down-regulated by tesaglitazar, the probes within the conserved TE module were also enriched with LINEs, SINEs, and LTRs in both brain areas and LINEs and SINEs in liver, as well as genes related to epigenetic modification. It is known that epigenetic modifications are important for host regulation of TEs [for example, see (Slotkin and Martienssen, 2007; Maze et al., 2011; Ponomarev et al., 2012; Xie et al., 2013; Du et al., 2016)]. We found several members of the Krüppel-associated box zinc finger protein family (Zfp445, Zfp612, Zfp551, Zfp52), in the conserved TE module in both brain areas (amygdala: green, PFC: tan), which are known to play a repressive role in transposition (Wolf et al., 2015). The chromatin remodeler, Chd1, and the lysine methyltransferase, Ehmt1, were also in the conserved TE module, and both have been shown to rather specifically repress retrotransposons due to their importance in heterochromatin formation (Maksakova et al., 2013; Green et al., 2017). Surprisingly, the change in expression after PPAR agonist treatment was the same for these repressive marks and the retrotransposons in these modules, which is opposite of what would be expected. However, it could be that the changes in the heterochromatin marks are a compensatory mechanism in response to changes in TE expression. In other words, if TE expression is increased, the cell will try to inhibit TE expression by increasing expression of repressive epigenetic factors.

The relationship between chromatin modifications and TE expression has also been observed in addiction. In the first study to suggest that TEs play a functional role in AUD, Ponomarev and colleagues used postmortem brains of alcoholic and control cases to show that chronic alcohol abuse resulted in transcriptional activation of LTR-containing transposons accompanied by DNA hypomethylation in their promoters (Ponomarev et al., 2012). It was shown in mice that repeated cocaine exposure decreases heterochromatic histone methylation (H3K9me3) binding and removes epigenetic silencing of LINE-1 elements in the nucleus accumbens (Maze et al., 2011). The role of PPARs in drug dependence is reviewed in (Le Foll et al., 2013), and is discussed below.

Actions of PPAR drugs have also been associated with epigenetic factors. There has been some exploration of the relationship between histone marks and PPARs/PPAR agonists. One study provides evidence that PPAR agonists can regulate epigenetic machinery (Li et al., 2017). In this study, pioglitazone (a PPARγ agonist) suppressed platelet-derived growth factor (PDGF)-stimulated cell proliferation by regulating Hdac1 *in vitro* (Li et al., 2017). There is also evidence of the inverse relationship, that is, that histone marks regulate PPARs and response to PPAR agonists. Hdac5 is dephosphorylated and translocated to the nucleus by fasting-induced rises in glucagon levels where it interacts with PPARα to promote its transcriptional activity, thereby mediating hepatic fatty acid oxidation by fasting and ER stress (Qiu et al., 2017). The histone H3 lysine 4 methyltransferase, Mll4/Kmt2d, directs overnutrition-induced murine steatosis via its coactivator function for PPARγ2, whereby overnutrition activates Abl1 kinase which phosphorylates PPARγ2, and hence has enhanced interaction with Mll4 (Kim et al., 2016). DNA methylation status of certain CpGs in CD4 + T cells is associated with the inflammatory response in people undergoing a 3-week fenofibrate treatment in the Genetics of Lipid Lowering Drugs and Diet Network (GOLDN) study (Yusuf et al., 2017). Furthermore, Dnmt1, a maintenance enzyme responsible for *de novo* methylation, regulates the methylation status of the PPARγ promoter in macrophages, where more Dnmt1 correlates with less PPARγ gene expression levels in atherosclerosis patients’ monocytes (Yu et al., 2016). Given the evidence for a relationship between chromatin marks, and both PPARs, and TE expression, the strong, clear relationship we observed from our network analysis between TEs, epigenetic regulators, and PPAR agonist-regulated genes supports our approach and serves as a starting point to guide future experiments exploring the epigenetic control and the biological significance of TEs.

Our findings have two potential implications and interpretations. One possibility is that PPAR agonists are regulating TE expression levels through a PPAR-dependent transcriptional response (i.e., binding of PPARs and associated complexes to DNA in TE promoter regions). Another possibility is that PPAR agonists are regulating TE expression levels in an indirect manner, possibly by modifying the epigenetic landscape. The coexpression of TEs, epigenetic regulators, and PPAR agonist-regulated genes suggests a means by which PPAR agonist administration could affect TE expression. Namely that fenofibrate could increase TE expression by a “passive” release of chromatin-mediated gene silencing, similarly to what was observed for repeated alcohol and cocaine exposure (Maze et al., 2011; Ponomarev et al., 2012). Another line of evidence supports a PPAR-independent effect of PPAR agonist administration on TE expression levels; fenofibrate and bezafibrate, but not tesaglitazar, up-regulated SINEs in PFC. Considering that all of the PPAR agonists in this study target PPARα, it is possible that some of the observed effects on TE expression are off target (meaning they are not mediated directly by PPAR activation). Other off target effects of PPAR agonists have been noted. For example, some of the insulin-sensitizing benefits of PPAR-γ agonists are due in part to their ability to block phosphorylation and not solely to their agonist activity (Choi et al., 2010). It will be important for future experiments to investigate the effects of PPAR agonist administration on TE expression in PPARα knockout mice to determine whether these effects on TE expression are mediated through PPARα.

In our study, the directionality of TE expression indicated that fenofibrate and bezafibrate up-regulated SINEs and LINEs in PFC and down-regulated them in liver. Superficially, the finding in the PFC seems contradictory (i.e., if PPAR agonists are anti-addictive they should *decrease*, not increase, TEs). We probed the transcriptional response at only a single time point. Therefore, it is possible that we are measuring a compensation for decreased TEs. It could also be that the effect of tesaglitazar was masked when analyzing the full list of differentially expressed genes and TEs, because a downregulation by tesaglitazar was linked to retrotransposons by the network analysis. Another interpretation is that, similarly to TEs, PPAR agonists have both beneficial and detrimental potential. Outside of their clinical uses of improving lipid and glycemic control, PPAR agonists have other therapeutic effects, but not unaccompanied by deleterious side effects. For example, PPAR agonists can be anti-inflammatory, hepatoprotective and caridoprotective (Staels et al., 2013; Youssef and Badr, 2004; Lee and Kim, 2015), but they can also lead to edema, weight gain, and hepatocellular and bladder carcinoma, to name a few (Turner et al., 2014) (Wright et al., 2014). Given the fact that increased expression of TEs can be a mark of genomic instability and lead to a number of diseases via genomic rearrangements and changes in gene expression regulation, perhaps some of PPARs deleterious effects are mediated though their up-regulation of TEs. Thus one consideration emerging from our study is that it might be prudent to test and observe a drug’s effects on TEs as an indication of possible negative effects on the patient, especially in cases where the drug is known to alter chromatin state.

In summary, we found evidence that PPAR agonist administration can modulate retrotransposon expression levels. This is one of only a few studies to demonstrate pharmacological regulation of TEs expression. We provide a basis for further study of the mechanisms and functional consequences of increased or decreased retrotransposon expression in brain and liver after PPAR agonist administration. Understanding whether PPAR agonists are mediating deleterious effects through retrotransposons could lead to the development of treatments with improved safety profiles. More broadly, this information could lead to a better understanding of and novel treatment strategies for the many psychiatric and neurological disorders that are associated with differential expression of retrotransposons.

## Author Contributions

LF, LZ, SW, CB, RH, and IP substantially contributed to the design of the work, the acquisition, analysis, and/or interpretation of data. LF drafted the work. RH and IP revised it critically. All authors provided final approval of the version to be published, and agreed to be accountable for all aspects of the work in ensuring that questions related to the accuracy or integrity of any part of the work are appropriately investigated and resolved.

## Conflict of Interest Statement

The authors declare that the research was conducted in the absence of any commercial or financial relationships that could be construed as a potential conflict of interest.

## Footnotes

1 http://support.illumina.com/array/downloads.html

2 http://genome.ucsc.edu/

3 http://www.webgestalt.org
