# Supplementary material for: Peroxisome Proliferator Activated Receptor Agonists Modulate Transposable Element Expression in Brain and Liver
Source: Front Mol Neurosci. 2018 Sep 19;11:331. doi: 10.3389/fnmol.2018.00331 (PMC6156381; doi:10.3389/fnmol.2018.00331)
Supplement: Supplementary file 7 [file Data_Sheet_1.docx]

Supplementary Material

**Peroxisome Proliferator Activated Receptor Agonists Modulate Transposable Element Expression in Brain and Liver**

Laura B. Ferguson, Ph.D.^1^, Lingling Zhang, Ph.D.^2^, Shi Wang, Ph. D.^2,3^, Courtney Bridges^1^, R. Adron Harris, Ph.D.^1^, Igor Ponomarev, Ph.D.^1*^

*** Correspondence:** Igor Ponomarev

Email: [ponomarev@utexas.edu](mailto:ponomarev@utexas.edu)

Laura Ferguson

Email: [laurazeavin@gmail.com](mailto:laurazeavin@gmail.com)

# Supplementary Figures and Tables

## Supplementary Figures

**Supplementary Figure 1.** Percentage of unannotated and protein-coding genes. The pie charts display the percentage of probes on the Illumina Mouse WG-6 microarray that detect protein-coding genes and the percentage that detect unannotated genes. We used information from UCSC Genome Browser to find probes that map to areas of the genome that contain transposable elements. The probes that target these areas could detect transposable elements (TEs). We found these probes are mostly unannotated, which supports the mapping technique.

**Supplementary Figure 2.** Overlap between modules of different tissue networks. We built and analyzed networks for each tissue individually and compared the co-expression patterns of each tissue’s network. The hypergeometric distribution was used to assess the significance of internetwork module overlap. The 13,623 probes that were common between amygdala, PFC, and liver networks were used in the network comparison. The number following the module name is the number of probes within that module. The number in the matrix represent the number of overlapping probes between the modules in the corresponding row and column. The color denotes the –log(hypergeometric p value), so the larger the number the more significant the overlap (the redder the color).


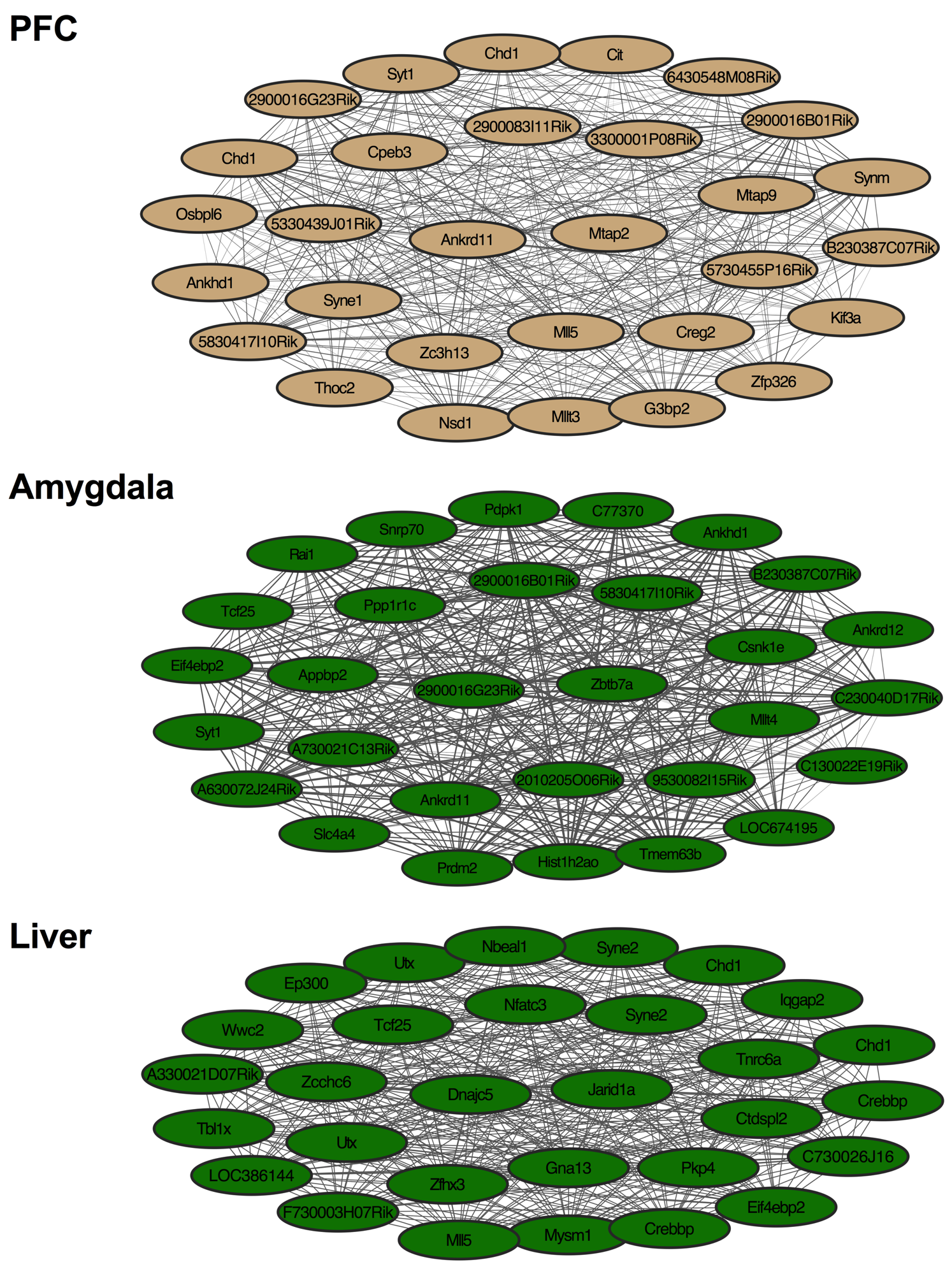


**Supplementary Figure 3.** Hub genes within the conserved TE modules. There was a highly significant overlap between the tan module in PFC, green module in liver and green module in amygdala (see Figure 4 in the main text and Figure S2 above). These modules share an enrichment of LINEs, SINEs, tesaglitazar down-regulated genes, neuronal genes (amygdala and PFC only) and genes known to be involved in epigenetic mechanisms (see Figure 3 and Table 2 in the main text). We used Cytoscape v3.2.1 to visualize the most interconnected probes within these modules, called hub genes. We calculated the intramodular connectivity (Kwithin in Table S4), which is a measure of how connected, or co-expressed, a given probe is with respect to the probes of a particular module. Displayed are the top 30 hub probes as determined by intramodular connectivity (the higher the intramodular connectivity, the more hub-like is the probe) for each of the conserved TE modules from each tissue.

**Most supplementary tables are in separate spreadsheets that can be found here:**

**https://www.frontiersin.org/articles/10.3389/fnmol.2018.00331/full#supplementary-material**

| **Amygdala** | **Beza** | **Feno** | **Tesa** |
| --- | --- | --- | --- |
| **SINE (all DEGs)** | 0.998 | 1.000 | 0.808 |
| **SINE (upregulated genes)** | 0.989 | 0.893 | 0.974 |
| **SINE (downregulated genes)** | 0.949 | 1.000 | 0.181 |
| **LINE (all DEGs)** | 0.627 | 0.998 | 0.986 |
| **LINE (upregulated genes)** | 0.985 | 0.980 | 0.999 |
| **LINE (downregulated genes)** | 0.097 | 0.964 | 0.536 |
| **LTR (all DEGs)** | 0.936 | 0.965 | 0.234 |
| **LTR (upregulated genes)** | 1.000 | 0.991 | 0.996 |
| **LTR (downregulated genes)** | 0.358 | 0.830 | 0.006 |
| **DNA (all DEGs)** | 0.905 | 0.110 | 0.369 |
| **DNA (upregulated genes)** | 0.667 | 0.654 | 0.716 |
| **DNA (downregulated genes)** | 1.000 | 0.030 | 0.210 |

**Table S3.** TEs in PPAR regulated genesets in amygdala. We previously characterized brain and liver gene expression profiles following an 8-day, systemic treatment with PPAR agonists [18]. Here, we characterized TE expression in the PPAR-regulated genesets (PPAR-regulated genes as those that were differentially expressed between PPAR agonist and saline treatment at p < 0.05). We used the hypergeometric test to determine if PPAR agonists regulated TEs more than chance level and used Bonferroni corrected p < 0.05 as a statistical threshold (bold p values are those that were significant at this threshold). We looked at enrichment in all differentially expressed genes (DEGs), upregulated genes, and downregulated genes as denoted by row name.
